# The power of vertical geolocation of atmospheric profiles from GNSS radio occultation

**DOI:** 10.1002/2016JD025902

**Published:** 2017-02-08

**Authors:** Barbara Scherllin‐Pirscher, Andrea K. Steiner, Gottfried Kirchengast, Marc Schwärz, Stephen S. Leroy

**Affiliations:** ^1^ Wegener Center for Climate and Global Change (WEGC) and Institute for Geophysics, Astrophysics, and Meteorology/Institute of PhysicsUniversity of GrazGrazAustria; ^2^ Zentralanstalt für Meteorologie und Geodynamik (ZAMG)ViennaAustria; ^3^ School of Engineering and Applied SciencesHarvard UniversityCambridgeMassachusettsUSA

**Keywords:** radio occultation, uncertainty, different vertical coordinates, atmospheric structure

## Abstract

High‐resolution measurements from Global Navigation Satellite System (GNSS) radio occultation (RO) provide atmospheric profiles with independent information on altitude and pressure. This unique property is of crucial advantage when analyzing atmospheric characteristics that require joint knowledge of altitude and pressure or other thermodynamic atmospheric variables. Here we introduce and demonstrate the utility of this independent information from RO and discuss the computation, uncertainty, and use of RO atmospheric profiles on isohypsic coordinates—mean sea level altitude and geopotential height—as well as on thermodynamic coordinates (pressure and potential temperature). Using geopotential height as vertical grid, we give information on errors of RO‐derived temperature, pressure, and potential temperature profiles and provide an empirical error model which accounts for seasonal and latitudinal variations. The observational uncertainty of individual temperature/pressure/potential temperature profiles is about 0.7 K/0.15%/1.4 K in the tropopause region. It gradually increases into the stratosphere and decreases toward the lower troposphere. This decrease is due to the increasing influence of background information. The total climatological error of mean atmospheric fields is, in general, dominated by the systematic error component. We use sampling error‐corrected climatological fields to demonstrate the power of having different and accurate vertical coordinates available. As examples we analyze characteristics of the location of the tropopause for geopotential height, pressure, and potential temperature coordinates as well as seasonal variations of the midlatitude jet stream core. This highlights the broad applicability of RO and the utility of its versatile vertical geolocation for investigating the vertical structure of the troposphere and stratosphere.

## Key Points

- Refractometric observations from RO provide independent information on altitude and pressure
- We estimate errors of RO‐derived temperature, pressure, and potential temperature and provide an empirical error model
- Example applications on tropopause and wind fields clearly reveal the power of vertical geolocation from RO measurements

## 1 Introduction

Precise knowledge and understanding of the thermodynamic state of the atmosphere and the global atmospheric circulation is crucial when investigating the physical climate system, climate variability and change [e.g., *Intergovernmental Panel on Climate Change*, 2013]. In this context, the upper troposphere and lower stratosphere (UTLS) region is particularly important because of different characteristics of the well‐mixed troposphere and the stably stratified stratosphere as well as their vertical coupling and interaction [e.g., *Gerber et al.*, 2012].

Observational evidence as well as modeling results have revealed that tropospheric warming and stratospheric cooling cause changes in tropopause characteristics [e.g., *Santer et al.*, 2003; *Austin and Reichler*, 2008]. Troposphere‐stratosphere transport of water vapor, which is crucial for stratospheric ozone chemistry as well as stratospheric radiative balance is thereby also affected [*Kirk‐Davidoff et al.*, 1999; *Forster and Shine*, 1999]. Changes of the atmospheric circulation system have also become evident as, for example, the expansion of the tropical belt [e.g., *Seidel et al.*, 2008; *Davis and Rosenlof*, 2012]. Since these changes in global atmospheric circulation are small, accurate, precise, and global data are required to get reliable information.

Investigations of the thermodynamic state of the atmosphere and global circulation are performed on different vertical coordinates depending on the purpose of the studies. Detection of specific atmospheric levels (such as the tropopause) and their changes with time can best be performed by using a vertical grid, which is independent from meteorological conditions. A vertical geopotential height grid or a mean sea level (MSL) altitude grid satisfy this requirement as they are both simply defined as height above the Earth's geoid. Note, however, that they are not the same because geopotential height accounts for local gravity effects while MSL altitude is a purely geometric vertical coordinate (see Appendix C). However, for simplifying analytical calculations in atmospheric science it is a distinct advantage to use thermodynamic coordinates such as isobaric coordinates (i.e., constant pressure surfaces), log‐pressure coordinates (i.e., pressure scaling like altitude), or isentropic coordinates (i.e., constant potential temperature surfaces) [*Salby*, 2012]. For example, the fact that under adiabatic conditions large‐scale air does not move along isohypsic surfaces (i.e., constant altitude), but is nearly tangential to isentropic surfaces, facilitates tracking the movement of bodies of air in this coordinate system.

Using a given observational data set on such different vertical coordinates requires atmospheric profiles of the very same (high) quality and known error characteristics. This is often problematic for satellite observations since precise knowledge of the satellite's antenna pointing and altitude attribution is needed which is challenging, for example, for passive limb sounding measurements [*Kiefer et al.*, 2007] and even more so for down looking radiometric measurements [*Elachi and van Zyl*, 2006].

In contrast, high‐vertical‐resolution atmospheric profiles of Global Navigation Satellite System (GNSS) radio occultation (RO) measurements provide information of measurement height and thermodynamic atmospheric variables. This space‐geodetic technique is based on refractometric measurements that are calibrated using atomic clocks. Temperature and pressure profiles of the UTLS are retrieved at the very same time with the same high quality. This is a distinct advantage for flexible use of data both on isohypsic and thermodynamic coordinates.

Detailed descriptions of the RO technique and retrieval of atmospheric variables are given by, e.g., *Melbourne et al.* [1994], *Kursinski et al.* [1997], *Steiner et al.* [2001], *Hajj et al.* [2002], and *Kuo et al.* [2004]. *Leroy* [1997] explicitly discussed the capability to retrieve geopotential height from RO measurements. However, so far, most studies used RO profiles as a function of MSL altitude [see *Anthes*, 2011; *Steiner et al.*, 2011]. Thermodynamic coordinates were used for some studies on upper tropospheric wind fields [*Scherllin‐Pirscher et al.*, 2014; *Verkhoglyadova et al.*, 2014] or stratospheric gravity waves [e.g., *Alexander et al.*, 2009].

In this paper we review and explain in detail the computation, use, and benefit of using different vertical coordinates from RO. The aim of this paper is to highlight that RO measurements provide precise information of atmospheric variables not only as a function of height/MSL altitude but also as a function of geopotential height, pressure/log‐pressure, and potential temperature. We provide uncertainty estimates for all these variables and demonstrate the utility of each coordinate by way of example.

## 2 RO Data and Uncertainty Estimates

### 2.1 RO Method and Data Characteristics

RO measurements are based on the exploitation of radio signals, which are operationally transmitted by GNSS satellites in an active limb sounding (occultation) geometry. On their way through the Earth's atmosphere, these signals are refracted until they are received on a satellite in low Earth orbit (LEO). Thermodynamic atmospheric variables can be retrieved from accurate and precise information of the satellites' orbits as well as measured excess phase path and amplitude information of the GNSS signals while they scan through the atmosphere [*Kursinski et al.*, 1997; *Steiner et al.*, 2001; *Hajj et al.*, 2002]. The measurement's position in the atmosphere, in particular of the vertical level of its tangent point (the point of closest approach of the signal propagation path between the GNSS and LEO satellite) is computed from the GNSS and LEO satellite positions and the observed Doppler shift assuming local spherical symmetry. It is therefore based on highly accurate geodetic measurements. The separation of the vertical grid and retrieved atmospheric parameters is performed early in the retrieval chain during bending angle and refractivity retrieval [e.g., *Kursinski et al.*, 1997]. In these early steps the height grid is established and adopted as the independent vertical coordinate on which the other atmospheric variables and their uncertainties depend. Information on measurement height and atmospheric variables such as temperature and pressure, the latter derived later in the chain from refractivity, are thus virtually independent from each other. This capability is unique for RO due to being a refractometric technique, in contrast to radiometric techniques such as passive infrared and microwave sounding.

Overall, the RO method has several distinct benefits: the technique provides measurements with very high accuracy (<1 K for individual profiles and <0.2 K for averages) and precision (<0.05 K) [*Kursinski et al.*, 1997; *Schreiner et al.*, 2007; *Anthes*, 2011; *Scherllin‐Pirscher et al.*, 2011a, 2011b; *Ladstädter et al.*, 2015]. Since RO measurements are self‐calibrating (due to the measurement principle), data from different satellites can be combined to a single long‐term stable climate record (better than 0.1 K consistency) [*Foelsche et al.*, 2011] and exhibit very low structural uncertainty in long‐term trends (<0.1 K per decade) [*Ho et al.*, 2009, 2012; *Steiner et al.*, 2013]. The horizontal resolution of about 60 km in the lower troposphere to about 300 km in the stratosphere [*Melbourne et al.*, 1994] is due to inherent along‐ray horizontal averaging of the method and allows applications at mesoscales to larger scales. The high vertical resolution (from about 0.1 km near the surface to about 1.5 km in the stratosphere [*Kursinski et al.*, 1997; *Gorbunov et al.*, 2004]) can be used to get information on selected levels such as the tropopause or the top height of the planetary boundary layer. Furthermore, RO is unique in its horizontal coverage. It is globally available from an increasing number of LEO satellites, evenly distributed across the Earth under essentially all‐weather conditions (clear and cloudy air), and asynoptic, over a full diversity of (local) times.

GNSS RO is an unusual remote sensing technique in that it has many qualities of space‐based atmospheric sounders and also of radiosondes. The qualities that GNSS RO has in common with space‐based sounders include global coverage and horizontal resolution, the latter typical of limb sounders. The qualities it has in common with radiosondes include vertical resolution and precision of temperature observation. Another quality that it has in common with radiosondes is accurate information on absolute position of air parcels in an Earth centered geodetic coordinate system. In contrast, radiometric (nadir) sounding techniques, e.g., passive infrared and microwave sounders, natively retrieve atmospheric variables such as temperature and water vapor on a pressure grid. The latter grid can only be converted into height using additional (model) information, since vertical geolocation is not an intrinsic part of radiometric sounding information itself.

### 2.2 Key Atmospheric Variables From RO

The fundamental atmospheric variable determining RO measurements is refractivity *N*, which is why RO is also termed a refractometric technique. Since RO is based on the refraction of GNSS signals and since GNSS uses the world geodetic system 1984 (WGS 84) reference coordinate system, retrieved atmospheric profiles from RO are intrinsically a function of height above the WGS 84 ellipsoid, termed ellipsoidal or geodetic height *h*. This ellipsoidal height, however, can be converted to height above the geoid and to geopotential height, without dependencies on atmospheric conditions (see also Appendix C).

#### 2.2.1 Height‐Related Information From RO

**Figure 1:**
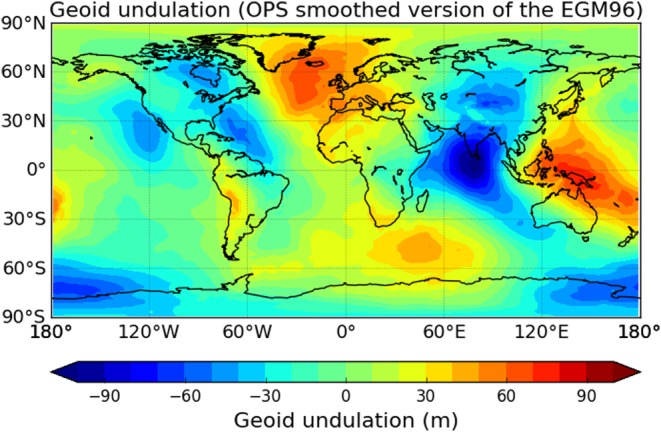
Global map of the geoid undulation with respect to the WGS 84 ellipsoid.

The difference between the Earth's ellipsoid and the geoid is referred to as geoid undulation *N*_u_, which is a function of longitude *λ* and latitude *φ*. In the Wegener Center (WEGC) occultation processing system (OPS), *N*_u_(*λ*,*φ*) is extracted from a smoothed version of the Earth Gravitational Model 1996 (EGM96) [*Lemoine et al.*, 1998]. It has a horizontal resolution of 2° × 2° in longitude and latitude. This horizontal resolution is chosen to approximately match with the horizontal resolution of RO measurements. Figure 1 shows that geoid undulation is within ±100 m in general, i.e., the difference between the geoid as the reference surface for the mean sea level and the Earth's rotational ellipsoid shape is rather small world wide. It is significant for accurate vertical geolocation at the 1 m level though. *N*_u_ is largest in the Indian Ocean (about −100 m), above Indonesia (about +80 m), and in the northern part of the Atlantic Ocean (about +70 m).

Extraction of *N*_u_(*λ*,*φ*) at mean tangent point location of the RO measurement allows to convert height above the WGS 84 ellipsoid *h* to height above the geoid *z*:

(1) $$z=h-N_{u}(\lambda ,\varphi ).$$

This height above the geoid is usually referred to as MSL altitude, altitude only, or orthometric height. Atmospheric profiles from RO measurements can therefore be referred to the Earth's geoid and are available as a function of altitude *z*, which is often the standard vertical coordinate for making RO data available to users.

The distribution of atmospheric mass is determined by gravity. Gravity force per unit mass *g* (i.e., gravitational acceleration) is the vertical derivative of the gravitational potential (or geopotential) Φ. It is defined as $\Phi (\lambda ,\varphi ,z)=\overset{z}{\underset{0}{\int }}g(\lambda ,\varphi ,z^{\prime })dz^{\prime }$ and involves contributions due to radial gravitation by the Earth's mass, centrifugal acceleration due to the Earth's rotation, and anisotropic effects [e.g., *Salby*, 2012]. This means that geopotential does not depend on atmospheric properties but is solely a function of position. A surface of constant geopotential thus only varies with longitude, latitude, and altitude.

Geopotential height *Z*(*λ*,*φ*,*z*) is defined as

(2) $$Z(\lambda ,\varphi ,z)=\frac{\Phi (\lambda ,\varphi ,z)}{g_{0}}=\frac{1}{g_{0}}\overset{z}{\underset{0}{\int }}g(\lambda ,\varphi ,z^{\prime })dz^{\prime },$$

where *g*_0_=9.80665 m s^−2^ is the standard gravitational acceleration [*National Oceanic and Atmospheric Administration et al.*, 1976]. As a reference surface generally mean sea level is chosen, i.e., the geoid. At this reference level altitude *z* and geopotential height *Z* are therefore both zero.

**Figure 2:**
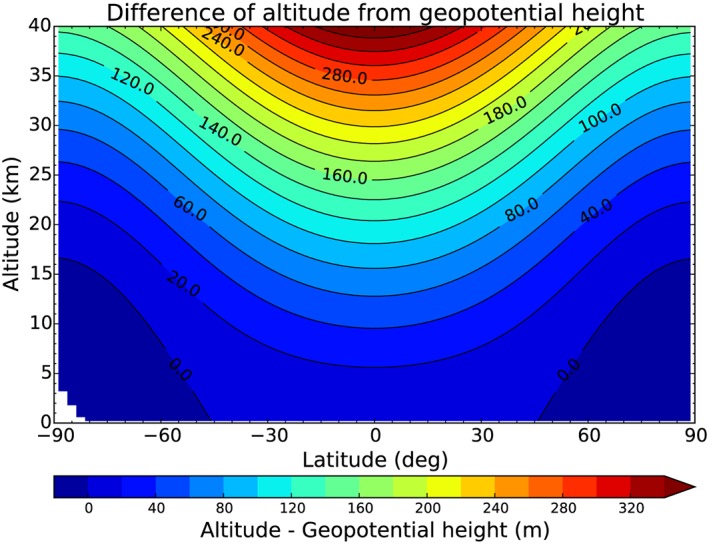
Difference between altitude *z* and geopotential height *Z* as a function of latitude and altitude.

Above the surface level, geopotential height is larger than altitude if local gravity is stronger than the standard gravitational acceleration and vice versa. Since gravity decreases from the pole (*g*_pole_≈9.82 m s^−2^) toward the equator (*g*_equator_≈9.79 m s^−2^) and since gravity decreases with altitude, largest differences are found at high altitudes and at equatorial latitudes as is illustrated in Figure 2. At 30 km, for example, the altitude exceeds the geopotential height by more than 200 m at low latitudes.

#### 2.2.2 From Refractivity to Derived Atmospheric Variables

Refractivity at microwave wavelengths in the neutral (uncharged) atmosphere mainly depends on thermodynamic conditions of the dry and the moist atmosphere. This relationship is given by the Smith‐Weintraub formula [*Smith and Weintraub*, 1953; *Kursinski et al.*, 1997],

(3) $$N(z)=k_{1}\frac{p(z)}{T(z)}+k_{2}\frac{e(z)}{T^{2}(z)},$$

with fairly high accuracy [*Aparicio and Laroche*, 2011; *Healy*, 2011], where the constants are *k*_1_=77.6 K Pa^−1^, *k*_2_=3.73 × 10^5^ K^2^ Pa^−1^, *p* is pressure (in hPa), *T* is temperature (in K), and *e* is partial pressure of water vapor (in hPa).

So‐called dry atmospheric parameters of RO measurements are obtained if the wet term of refractivity (i.e., the second term on the right‐hand side of equation (3)) is neglected. In this case total refractivity is considered as being dry‐air refractivity only, and dry density, dry pressure, and dry temperature differ from real atmospheric conditions as the real atmosphere contains atmospheric water vapor [*Scherllin‐Pirscher et al.*, 2011a]. In regions, where moisture is not negligible, dry density and dry pressure are higher than their physical equivalents; dry temperature is lower than physical temperature. A detailed discussion of the representativeness of dry atmospheric variables for physical variables is given in *Scherllin‐Pirscher et al.* [2011a].

If water vapor is not negligible (i.e., mainly in the lower troposphere) and physical atmospheric variables are of interest, background information is needed to separate the dry and moist contributions of refractivity [see e.g., *Kursinski et al.*, 1995; *Healy and Eyre*, 2000]. This background information allows the derivation of moist atmospheric variables such as specific humidity, water vapor mixing ratio, or water vapor pressure, together with temperature.

At WEGC, physical atmospheric variables are obtained from optimal estimation using auxiliary temperature and humidity data [*Schwärz et al.*, 2016, pp. 142–145]. These auxiliary data are obtained from European Centre for Medium‐Range Weather Forecasts (ECMWF) short‐range forecasts (24 h or 30 h forecast fields) in order to keep independence from ECMWF analyses or reanalyses, which contain RO information from data assimilation. In a first step, prescribed temperature and humidity profiles and their uncertainties are used to get a first estimate of RO‐derived humidity and temperature profiles and their uncertainties, respectively. In a second step, the final temperature and humidity profiles are calculated with optimal estimation based on inverse variance weighting between RO‐derived and background profiles. This approach, facilitating uncertainty propagation and algorithmic insight, is different from the 1D‐Var cost function minimization algorithm [e.g., *Healy and Eyre*, 2000] applied at most RO processing centers. The retrieval performance is very similar. Detailed information on the WEGC moist air retrieval has been presented by *Li et al.* [2016], and a manuscript is in preparation for submission to Journal of Geophysical Research—Atmospheres.

In this study, we use physical atmospheric variables. We calculate potential temperature *θ* using pressure *p* (in hPa), temperature *T* (in K), and specific humidity *q* (in kg kg^−1^). Because potential temperature also somewhat depends on atmospheric humidity [*Jacobson*, 1999; *Lee and Koh*, 2014], we calculate virtual potential temperature,

(4) $$\theta (z)=T_{v}(z){\left(\frac{p_{0}}{p(z)}\right)}^{\kappa (1-0.251q(z))},$$

where *T*_v_=*T*(1 + 0.608*q*) is virtual temperature, *p*_0_=1000 hPa is standard pressure, and *κ* = *R*/*c*_*p*_=0.286 is the adiabatic constant (*R* = 287.05 J kg^−1^ K^−1^ is the gas constant for dry air, and *c*_*p*_=1004.67 J kg^−1^ K^−1^ is specific heat of dry air at constant pressure at 298 K) [*Jacobson*, 1999]. For simplicity virtual potential temperature is referred to simply as potential temperature subsequently; the difference from dry‐air potential temperature $\theta (z)=T(z){\left(\frac{p_{0}}{p(z)}\right)}^{\kappa }$ is very small (<0.1%) except for significant moisture content (>2 g kg^−1^).

### 2.3 Uncertainty Estimates

For further considerations, we assume there is no uncertainty in ellipsoidal height *h* but attribute all uncertainty to refractivity *N*. Note that this is actually not entirely true because retrieved atmospheric profiles from RO and related height information are both contaminated with measurement errors. However, it is possible to “transfer” the uncertainty in height to the uncertainty in atmospheric variables and use height as an independent variable (see *Syndergaard* [1999] for more details).

#### 2.3.1 Uncertainty in MSL Altitude and Geopotential Height

Given perfect knowledge of *h*, uncertainty of MSL altitude *z* is limited only by the accuracy of the geoid undulation *N*_u_. The choice of the Earth geoid model and its horizontal resolution are therefore key factors, which determine the accuracy of MSL altitude. The EGM96 geoid [*Lemoine et al.*, 1998] and improved successors such as the EGM2008 [*Pavlis et al.*, 2011] are generally consistent within 1 m so that the MSL altitude uncertainty is estimated at the submeter level.

The accuracy of geopotential height *Z* is additionally limited by the accuracy of the Earth gravity model. A comparison based on employing two independent gravity models (described in detail in Appendix A) confirms that the uncertainty of geopotential height from adequate gravity modeling is smaller than 1–2 m up to 35 km.

**Figure A1:**
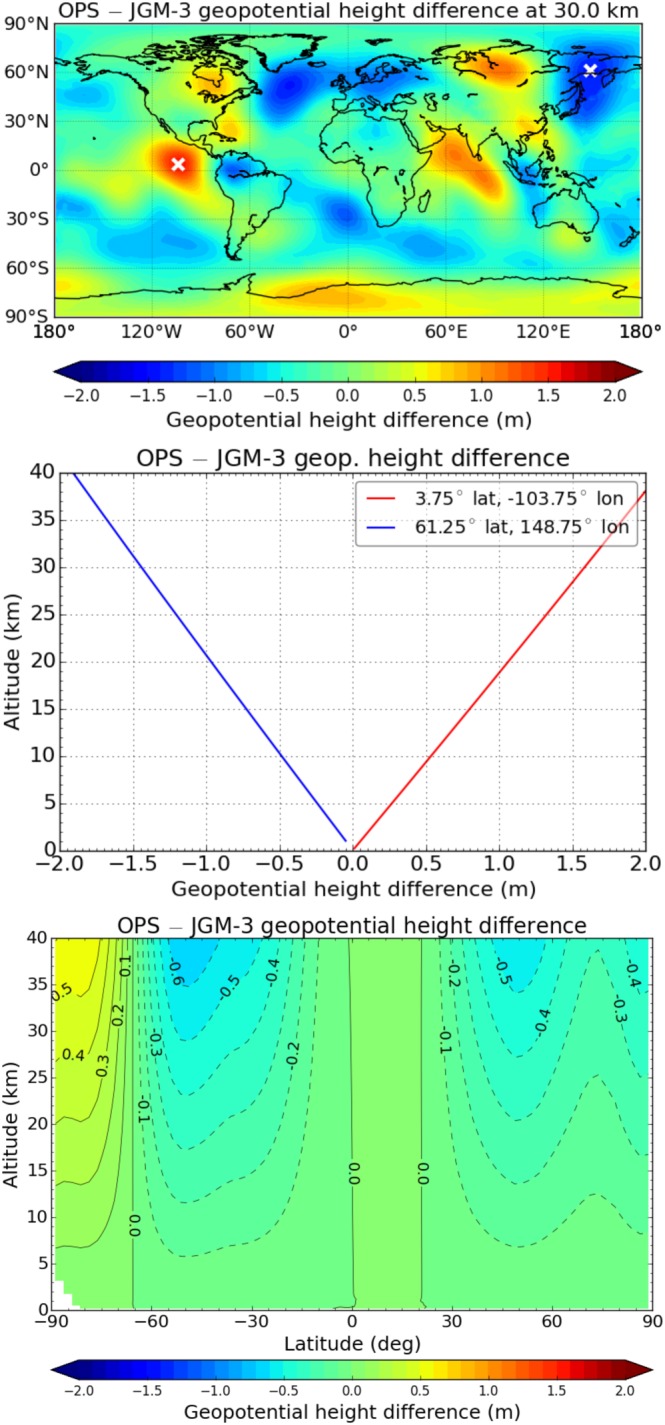
(top) Global map of the difference between OPS and JGM‐3 geopotential height at 30 km, (middle) vertical difference profiles at locations, where maximum positive (red) and negative (blue) differences are found and shown as function of altitude, and (bottom) zonal mean difference as a function of latitude and altitude. Geopotential height of the JGM‐3 model is obtained using 12 degrees of spherical harmonics. The white crosses in Figure A1 (top) denote the locations, where maximum positive and negative differences are found.

Due to the very small differences close to the surface (Figure A1, middle), we see confirmed that the uncertainty of MSL altitude as provided by the WEGC's OPS is much smaller than 1 m, in fact <0.1 m at the locations illustrated.

#### 2.3.2 Uncertainty in Atmospheric Profiles

An empirical error analysis of *Scherllin‐Pirscher et al.* [2011b] provided information on vertical, latitudinal, and seasonal characteristics of observational errors of individual profiles of RO bending angle, refractivity, and dry atmospheric variables. Here we extend this study by providing an uncertainty description also for physical atmospheric variables retrieved with the WEGC OPSv5.6 [*Schwärz et al.*, 2016, pages 142–145].

**Figure 3:**
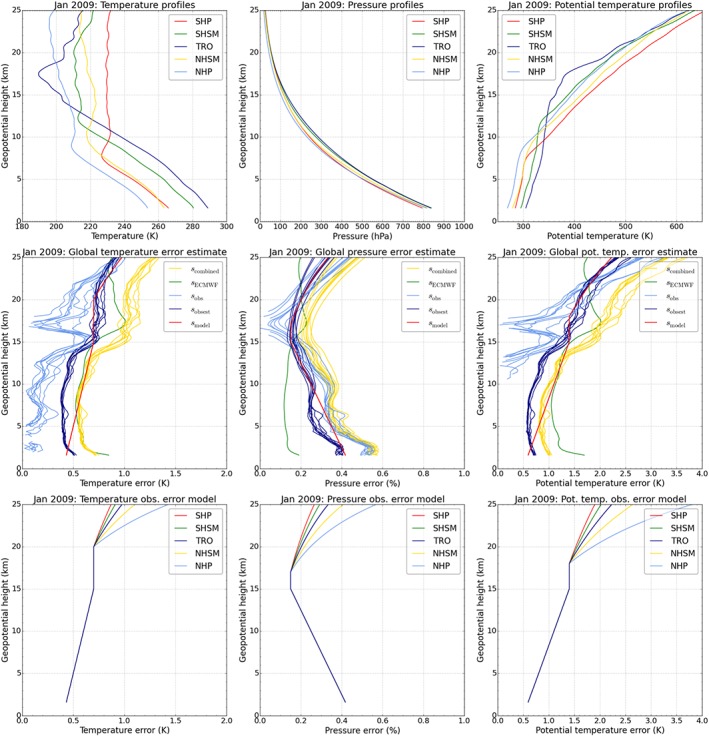
(top row) Individual RO atmospheric profiles for different regions, Southern/Northern Hemisphere polar (SHP/NHP), Southern/Northern Hemisphere subtropics and midlatitudes (SHSM/NHSM) and tropics (TRO); (middle row) global error estimates; (bottom row) and observational error model for the different regions, for temperature, pressure, and potential temperature (left to right) for January 2009. In Figure 3 (middle row), the bundles of even‐colored profiles represent the ensemble of RO satellites included, comprising the six F3C satellites (Formosa satellite mission #3/Constellation Observing System for Meteorology, Ionosphere, and Climate), GRACE (Gravity Recovery and Climate Experiment), and SAC‐C (Satélite de Aplicaciones Cientificas‐C).

Figure 3 illustrates this description, and Table 1 (observational error block) summarizes error model parameters for the key variables of interest. Observational error estimates of individual profiles of temperature, pressure, and potential temperature (Figure 3, top row) are based on the comparison of retrieved atmospheric profiles from RO to colocated profiles from ECMWF analysis fields. The standard deviation of the RO minus ECMWF difference profiles reveals information on the combined error *s*_combined_. A reasonable estimate of the observational error (*s*_obsest_) can be obtained from scaling the combined observational error with $1/\sqrt{2}$ [*Scherllin‐Pirscher et al.*, 2011b].

**Table 1:** Estimated Error Model Parameters for the Observational Error, Sampling Error, and Systematic Error for Temperature *T* (in K), Pressure *p* (in %), and Potential Temperature *θ* (in K)^a^

	*z*_Ttop_	*z*_Sbot_	*s*_00_	Δ*s*_0_	*q*_0_	*b*	*H*_S0_	Δ*H*_S_
*Observational Error*								
*T*	15.0 km	20.0 km	0.7 K	0.0 K	0.02 km^*b*^	−1.0	15.0 km	−8.0 km
*p*	15.0 km	17.0 km	0.15%	0.0%	−0.02 km^*b*^	−1.0	10.0 km	−4.0 km
*θ*	15.0 km	18.0 km	1.4 K	0.0 K	0.06 km^*b*^	−1.0	15.0 km	−8.0 km
*Sampling Error*								
*T*	10.0 km	25.0 km	0.3 K	1.5 K	−0.01 km^*b*^	−1.0	25.0 km	0.0 km
*p*	10.0 km	25.0 km	0.15%	1.2%	−0.01 km^*b*^	−1.0	25.0 km	0.0 km
*θ*	10.0 km	25.0 km	0.5 K	2.5 K	−0.01 km^*b*^	−1.0	25.0 km	0.0 km
*Systematic Error*								
*T*	15.0 km	20.0 km	0.1 K	0.05 K	−0.01 km^*b*^	−1.0	11.0 km	0.0 km
*p*	15.0 km	20.0 km	0.1%	0.05%	−0.01 km^*b*^	−1.0	11.0 km	0.0 km
*θ*	15.0 km	20.0 km	0.15 K	0.075 K	−0.01 km^*b*^	−1.0	11.0 km	0.0 km

a These model parameters are defined in Appendix B. Note that the unit of *q*_0_ depends on the parameter *b*, which is given in the column to the right.

A better estimate of the observational error (*s*_obs_) can be obtained from subtracting the estimated ECMWF analysis error *s*_ECMWF_ from the combined error in terms of variances (see *Scherllin‐Pirscher et al.* [2011b] for details),

(5) $$s_{\mathrm{obs}}=\sqrt{s_{\mathrm{combined}}^{2}-s_{\mathrm{ECMWF}}^{2}}.$$

While the global mean ECMWF temperature error is provided by the ECMWF, we obtain ECMWF model errors of pressure and potential temperature by applying empirically derived conversion factors (see *Scherllin‐Pirscher et al.* [2011b] for details). *Scherllin‐Pirscher et al.* [2011b] found that relative pressure errors (in %) are about 0.23 times the absolute temperature errors (in K). On top of this we find that potential temperature errors (in K) are about twice as large as temperature errors (in K). Figure 3 (middle row) illustrates these four estimated error profile types as well as the resulting observational error model for January 2009. Detailed information on the RO observational error model is given in Appendix B.

Table 1, upper part, summarizes the parameters of the observational error model for temperature, pressure, and potential temperature. The parameters have been empirically derived by fitting the model to RO data from different satellites from different years and inspecting various atmospheric situations. In the stratosphere, these parameter settings are identical to *Scherllin‐Pirscher et al.* [2011b]. In the troposphere, however, the observational error model is adapted due to the influence of background information in the retrieval of physical variables below 15 km. We account for the fact that tropospheric temperature and potential temperature errors decrease about linearly from 15 km toward the boundary layer, while tropospheric pressure increases about linearly (Figure 3, bottom panels). We note that the detailed shape of the observational error of physical atmospheric parameters in the troposphere, and therefore the values of the model parameters, depends on the moist air retrieval and on the choice of the background information. However, with adjustment of model parameter values as needed, the error model is also applicable to RO data sets from other data centers.

#### 2.3.3 Uncertainty in Climatological Fields

Climatological fields of atmospheric variables are affected by random statistical errors, (residual) sampling errors, and systematic errors. *Scherllin‐Pirscher et al.* [2011a] provided an empirical‐analytical error model for all these types of errors for bending angle, refractivity, and dry atmospheric variables. Here we extend that study by providing model parameters also for the physical atmospheric variables temperature, pressure, and potential temperature.

Statistical errors *s*_statErr_ exhibit random error characteristics. Therefore, they decrease with an increasing number of averaged profiles *N*_prof_. Using the observational error model *s*_model_ described in Appendix B, statistical errors are modeled by

(6) $$s_{\mathrm{statErr}}=\frac{s_{\mathrm{model}}}{\sqrt{N_{\mathrm{prof}}}}.$$

The formulations of the sampling error model and the systematic error model are also based on the generic error model formulation described in Appendix B. The model parameters for these error models are summarized in Table 1, middle and lower part.

For the sampling error as well as for the systematic error, we accounted for latitudinal and seasonal variations of the error in the UTLS core region in terms of the error magnitude [*Scherllin‐Pirscher et al.*, 2011a]. Increased systematic and sampling errors are modeled at high latitudes. Furthermore, the errors are larger in hemispheric winter than in hemispheric summer. Detailed information on this error modeling is given in Appendix B.

Sampling error estimates can reasonably be obtained using reference data, which adequately represent actual spatial and temporal atmospheric variability [see, e.g., *Pirscher et al.*, 2007; *Foelsche et al.*, 2008]. Subtracting the sampling error estimates from mean climatological fields leaves a residual sampling error. *Scherllin‐Pirscher et al.* [2011a] found significantly reduced magnitudes of the residual sampling error, amounting to about 30% of the original one. We adopt this reduction factor when modeling the residual sampling error *s*_resSamplErr_=0.3*s*_samplErr_ of climatological fields of temperature, pressure, and potential temperature.

Knowledge of these three individual (uncorrelated) climatological error components allows the computation of the total climatological error *s*_totErr_ in the form

(7) $$s_{\mathrm{totErr}}=\sqrt{s_{\mathrm{statErr}}^{2}+s_{\mathrm{resSamplErr}}^{2}+s_{\mathrm{sysErr}}^{2}}.$$

If the sampling error is not subtracted from the climatology, the total climatological error contains the full sampling error *s*_samplErr_ instead of the residual one.

**Figure 4:**
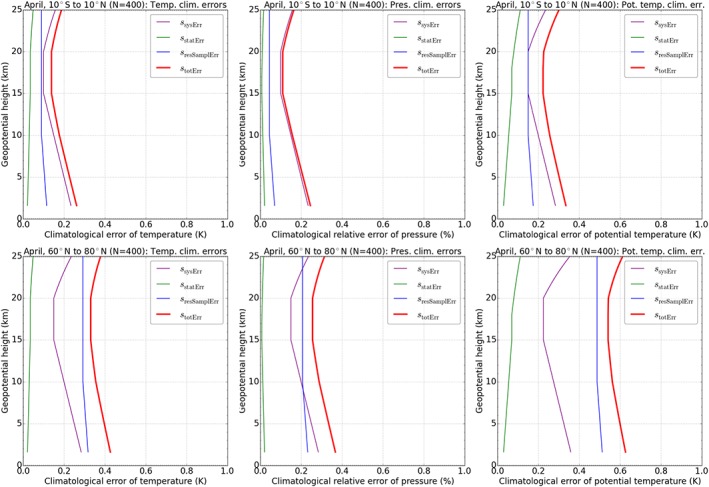
Climatological error model results for temperature, pressure, and potential temperature (left to right) at (top row) tropical latitudes 10°S to 10°N and (bottom row) high northern latitudes 60°N to 80°N as found for April conditions. Systematic error is shown in purple, statistical error in green, residual sampling error in blue, and total climatological error in red.

Figure 4 illustrates the individual error components as well as the total climatological error for April conditions at low latitudes as well as at high northern latitudes. The systematic error clearly dominates the total climatological error at low latitudes. The dominant error component at high latitudes, on the other hand, is the residual sampling error. This is due to larger atmospheric variability caused by tropospheric weather variations (e.g., synoptic systems and fronts) and stratospheric gravity waves.

## 3 Use of Different Vertical Coordinates From RO

### 3.1 Atmospheric Structure in Isohypsic, Isobaric, and Isentropic Coordinates

Vertical profiles of atmospheric variables from RO measurements are usually available on isohypsic coordinates. Isohypsic coordinates refer to a vertical coordinate that depends only on geometric position with respect to Earth's rotating frame and not on atmospheric state, two examples being mean sea level altitude and geopotential height. However, as we introduced and prepared above, it is possible to use atmospheric profiles from RO alternatively also on other vertical coordinates. We term this the power of vertical geolocation from RO.

In particular, the availability of accurate and precise pressure and potential temperature profiles (which are retrieved virtually independently from height; see section 2.1) allows the reliable alternative use of these thermodynamic variables as vertical coordinates. This is done through simple mapping from *p*(*z*), *θ*(*z*) to *z*(*p*), *z*(*θ*) or from *p*(*Z*), *θ*(*Z*) to *Z*(*p*), *Z*(*θ*) and interpolation to conveniently prescribed vertical grids of constant pressure levels or constant potential temperature levels. All atmospheric variables from RO measurements can therefore be readily used as well on isobaric and isentropic surfaces.

**Figure 5:**
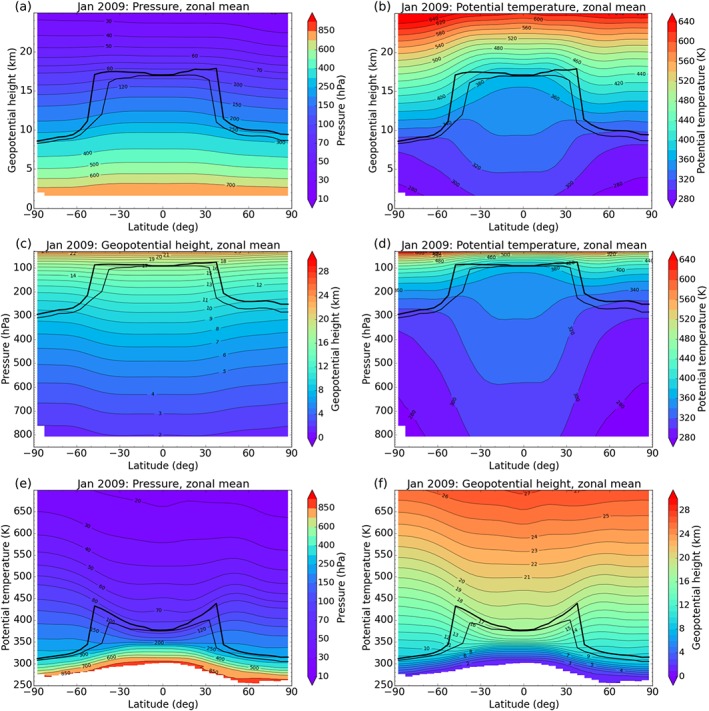
(top row) Pressure and potential temperature as a function of geopotential height, (middle row) geopotential height and potential temperature as a function of pressure, and (bottom row) pressure and geopotential height as a function of potential temperature for January 2009. The heavy and light (black) lines indicate the location of the cold point and lapse rate tropopause, respectively.

For exploiting the value of vertical geolocation and for inspecting the implications of using different vertical coordinates, we calculate sampling error‐corrected monthly mean 5° zonal mean fields of temperature, pressure, potential temperature, and geopotential height. Figure 5 shows example results for January 2009. Figures 5a and 5b show pressure and potential temperature as a function of geopotential height. These fields are obtained using 200 m vertical spacing of geopotential height from 1.6 km to 25.0 km (i.e., essentially above the planetary boundary layer up to the lower stratosphere) yielding 118 geopotential height levels.

The monotonic and nearly exponential decrease of pressure with height justifies the possibility of the log‐pressure coordinate *z*_*p*_:

(8) $$z_{p}(z)=H_{0}\ln\left(\frac{p_{s}}{p(z)}\right),$$

where *p*_s_=1013.25 hPa is the standard surface pressure and *H*_0_=7000 m is a standard scale height for the troposphere and stratosphere. Therefore, a convenient vertical pressure grid can be obtained from calculating pressure levels from an equidistant 200 m standard pressure altitude grid spanning 1.6 km to 25.0 km (118 pressure levels). Following this procedure and using the inverse relation $p=p_{s}\exp(-z_{p}/H_{0})$, we obtain a nonequidistant pressure grid from about 806 hPa to 28 hPa corresponding to an equidistant log‐pressure grid. Figures 5c and 5d show geopotential height and potential temperature fields as a function of this pressure grid.

In this context, the geopotential height and pressure coordinates chosen in Figures 5b and 5d illustrate the typical behavior of isentropic (potential temperature) surfaces. The vertical gradient of potential temperature (d*θ*/d*z*) is a measure for atmospheric stability. Since atmospheric stability is low in the troposphere, the vertical separation between isentropic surfaces is large. At low latitudes, for example, the separation between the 340 K and 360 K isentropes is larger than 5 km (Figure 5b). In the stratosphere, however, stability is high and the separation between isentropic surfaces is significantly smaller.

We account for these tropospheric‐stratospheric differences when choosing a suitable vertical grid for fields on isentropic surfaces. To capture tropospheric characteristics, we chose a 1 K potential temperature grid from 250 K up to 400 K. Above 400 K, we chose a 5 K grid, which is approximately equivalent to the 200 m geopotential height grid [*Crutzen and Freie*, 1997; *Knox*, 1998]. This yields 211 potential temperature levels in total. Figures 5e and 5f show pressure and geopotential height fields on this vertical potential temperature grid.

In order to estimate the sensitivity of potential temperature to atmospheric humidity, we also alternatively calculated mean fields of potential temperature of dry air assuming absence of water vapor (i.e., *q* = 0 in equation (4)). As expected, for our example climatological applications in the UTLS (discussed next in sections 3.2 and 3.3 below), we found that the moisture dependence is negligible. In general, small differences (up to ∼1 K) will only occur in lower troposphere regions with substantial humidity while the differences at tropopause levels are below 0.001 K.

Note that on individual profile basis the construction of the grids of log‐pressure and potential temperature depends on the vertical resolution of these profiles, since smooth profiles will contain less fluctuations than higher‐resolved ones. In averaged profiles the grid's level spacing will be very robust, however, since it will converge to the mean thermodynamic structure.

Overall the diversity of possible views of the atmospheric structure illustrated in Figure 5 well signals the versatility available for applying RO in different atmospheric studies.

### 3.2 Example Application: Tropopause Parameters From RO

High vertical resolution and high accuracy and precision, as provided by RO, are important when investigating characteristics of atmospheric key levels (e.g., tropopause height) or the width of specific layers (e.g., the tropical tropopause layer). Several studies already demonstrated the high quality and value of tropopause parameters obtained from RO measurements [e.g., *Schmidt et al.*, 2004; *Son et al.*, 2011; *Rieckh et al.*, 2014]. We therefore use this as one example application within this study and demonstrate the value of different vertical views, all with high accuracy and precision.

We calculated tropopause geopotential height from sampling error‐corrected monthly mean 5° zonal mean temperature climatologies from 2009, applying the lapse rate tropopause definition of *World Meteorological Organization* [1957]. Tropopause pressure and tropopause potential temperature are found through simple mapping of geopotential height to the other atmospheric parameters as introduced above. This yields the location of the tropopause in isobaric and isentropic coordinates. For context, recall that Figure 5 clearly revealed a mainly tropospheric view when plotting isobaric coordinates linearly between 850 hPa and 30 hPa (Figures 5c and 5d) but a mainly stratospheric view when plotting isentropic coordinates linearly between 250 K and 700 K (Figures 5e and 5f).

**Figure 6:**
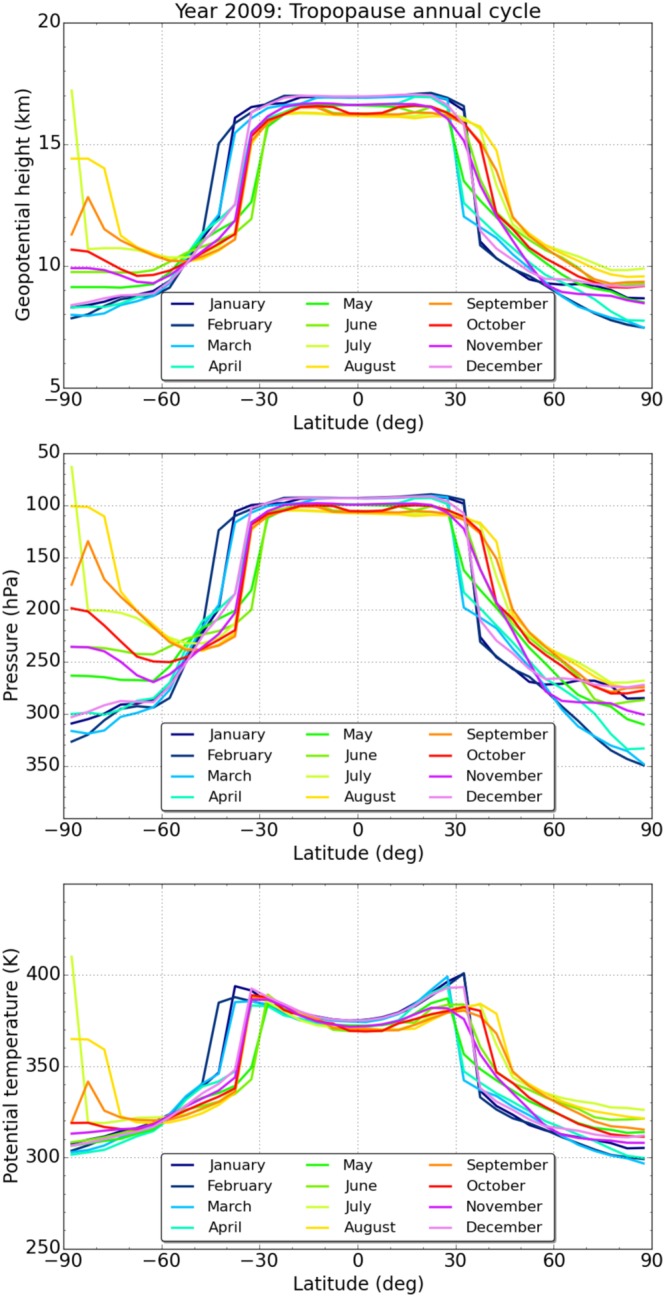
(top) Geopotential height, (middle) pressure, and (bottom) potential temperature of the lapse rate tropopause as a function of latitude for January to December 2009 (different colors).

Figure 6 shows seasonal characteristics of the lapse rate tropopause on isohypsic, isobaric, and isentropic coordinates. At low latitudes the tropopause is found between 16 km and 17 km for geopotential height and between 90 hPa and 110 hPa for pressure in all months. For potential temperature it increases from approximately 370 K at the equator to about 400 K at 30°S/N. Tropopause geopotential height, pressure, and potential temperature decrease toward high latitudes. At high northern latitudes they reach between 8 km and 10 km, 350 hPa and 260 hPa, and 300 K and 330 K, respectively. Lowest tropopauses (for all parameters) are found here at the winter‐to‐spring transition, February and March. This occurs for the same months at high southern latitudes, where this is the summer‐to‐fall transition and where annual variability is the largest worldwide. However, note that very high tropopauses in Southern Hemisphere winter are partly caused by the deficiency of the lapse rate tropopause definition, which is not well suited to adequately identify tropopauses in very cold stratospheric conditions as found in the southern polar winter [*Zängl and Hoinka*, 2001].

Uncertainty of these tropopause characteristics can be obtained from the climatological error estimates discussed in section 2.3.3. We discuss this here as an example for the low latitudes within 30° of the equator. Since the tropopause parameter estimates are based on sampling error‐corrected temperature fields, they only contain the residual sampling error. At low latitudes, this error component amounts to about 0.1 K for temperature, 0.05% for pressure, and 0.15 K for potential temperature. The systematic error of low latitude tropopause parameters is estimated at 0.1 K for temperature, 0.1% for pressure, and 0.15 K for potential temperature. Using RO data from all missions and assuming 1500 profiles per 5° latitude band per month (typically available as of 2007), the corresponding statistical error amounts to <0.025 K for temperature, <0.005% for pressure, and <0.05 K for potential temperature. This adds up to a total error of tropopause parameters at low latitudes of less than 0.15 K for temperature, about 0.1% for pressure (≈0.1 hPa), and about 0.2 K for potential temperature. This underpins the excellent quality of RO measurements for analyzing vertical level surfaces of interest in the UTLS.

### 3.3 Climatological Wind Fields

*Scherllin‐Pirscher et al.* [2014] and *Verkhoglyadova et al.* [2014] showed that climatological wind fields can be inferred in the extratropics from RO geopotential heights of pressure levels. They showed that RO derived geostrophic and gradient winds clearly capture all of the main wind features. The analysis of such wind fields based on RO data is therefore chosen as another example application in this study, to demonstrate the power of vertical geolocation in particular for the thermodynamic coordinates.

*Verkhoglyadova et al.* [2014] found the sampling error and ageostrophy to be the dominating error sources for wind fields. Accounting for the sampling error (i.e., subtracting the estimated sampling error) before calculating wind fields, *Scherllin‐Pirscher et al.* [2014] found that biases are, in general, smaller than 2 m s^−1^. Larger biases (up to 10 m s^−1^) are caused by the geostrophic/gradient wind approximation, which is violated close to the subtropical jet and at high latitudes. Residual sampling errors and systematic errors from RO are found comparatively negligible.

Horizontal geostrophic wind $\vec{v}$ on isobaric coordinates is defined as

(9) $${\vec{v}}_{p}=\frac{1}{f}\left(\vec{k}\times \nabla_{p}\Phi \right),$$

while applying a coordinate transformation from isobaric to isentropic coordinates yields

(10) $${\vec{v}}_{\theta }=\frac{1}{f}\left(\vec{k}\times \nabla_{\theta }\Psi \right),$$

where f=2Ωsinφ is the Coriolis parameter (Ω = 7.2921 × 10^−5^ rad s^−1^ is the Earth's rotation rate and *φ* the geodetic latitude), $\vec{k}$ is the vertical unit vector, ∇_*p*_ and ∇_*θ*_ are the horizontal gradients on an isobaric and isentropic surface, respectively, Φ = *g*_0_ *Z* is the geopotential (*g*_0_=9.80665 m s^−1^ being the standard gravity), and Ψ = Φ + *c*_*p*,m_ *T*_v_ is the Montgomery potential, where *c*_*p*,m_=*c*_*p*_(1 + 0.859*q*) is the specific heat of moist air at constant pressure [*Jacobson*, 1999].

To compute climatological RO winds, we first calculate monthly mean climatological fields of (i) geopotential at pressure levels and (ii) Montgomery potential at potential temperature levels, at 5° latitude times 5° longitude resolution following *Scherllin‐Pirscher et al.* [2014]. Subsequently, based on equations (9) and (10) we derive monthly mean geostrophic wind fields (outside the tropics) from the sampling error‐corrected climatological fields. To investigate the vertical characteristics of climatological wind, we then calculated the maximum column wind speed from scanning each wind column (5° × 5° bin) from 500 hPa to 50 hPa above the tropopause, similar to *Strong and Davis* [2005] and *Davis and Birner* [2013]. If the maximum column wind speed is found greater than 20 m s^−1^, geopotential height, pressure, and potential temperature are extracted at the vertical level of maximum wind speed.

**Figure 7:**
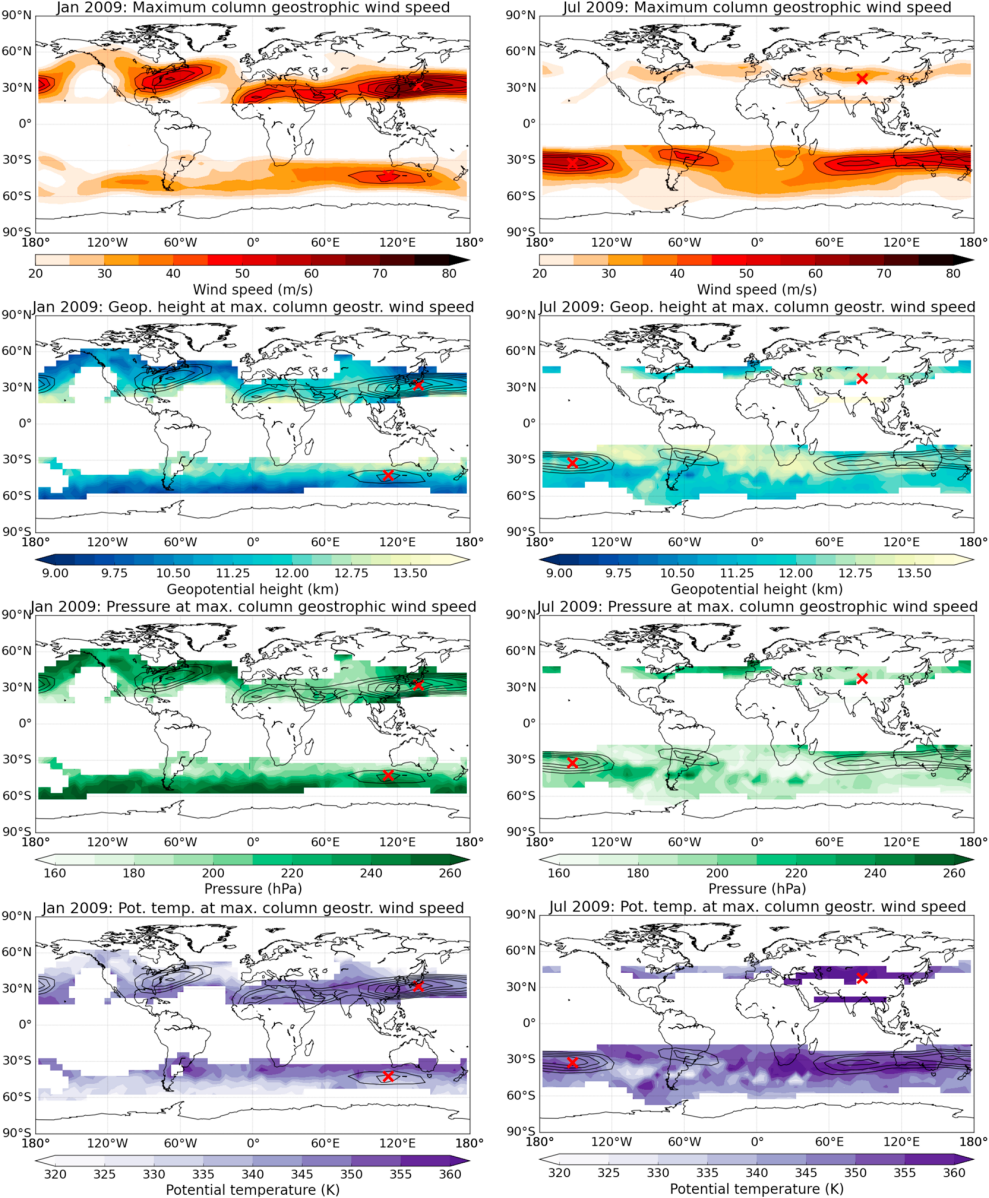
(first row) Maximum column wind speed and (second row) geopotential height, (third row) pressure, and (fourth row) potential temperature at the level where maximum column wind speed has been found for January 2009 (left column) and July 2009 (right column). Black contour lines indicate maximum wind speeds higher than 40 m/s. The two red crosses indicate the top maximum wind speed between 20°N and 50°N and 20°S and 50°S, respectively.

Figure 7 shows representative results for January and July 2009. Geostrophic wind speeds (Figure 7, first row) are largest in the winter hemisphere at midlatitudes, where maximum monthly mean wind speed reaches about 70 m s^−1^ in the Northern Hemisphere (January 2009) and about 60 m s^−1^ in the Southern Hemisphere (July 2009). Geopotential height, pressure, and potential temperature vary widely between about 9 km and 13 km, 180 hPa and 250 hPa, and 330 K and 360 K, respectively. Even in regions with wind speeds higher than 40 m s^−1^, the level of maximum column wind speed varies considerably in geopotential height, pressure, and potential temperature.

To investigate the horizontal and vertical location of tropospheric/tropopause region wind speed maxima and their annual cycle, we searched for the location of the absolute maximum of maximum column wind speed, i.e., the jet stream core, in the Northern and Southern Hemispheres between 20° and 50° latitude (red crosses in Figure 7).

**Figure 8:**
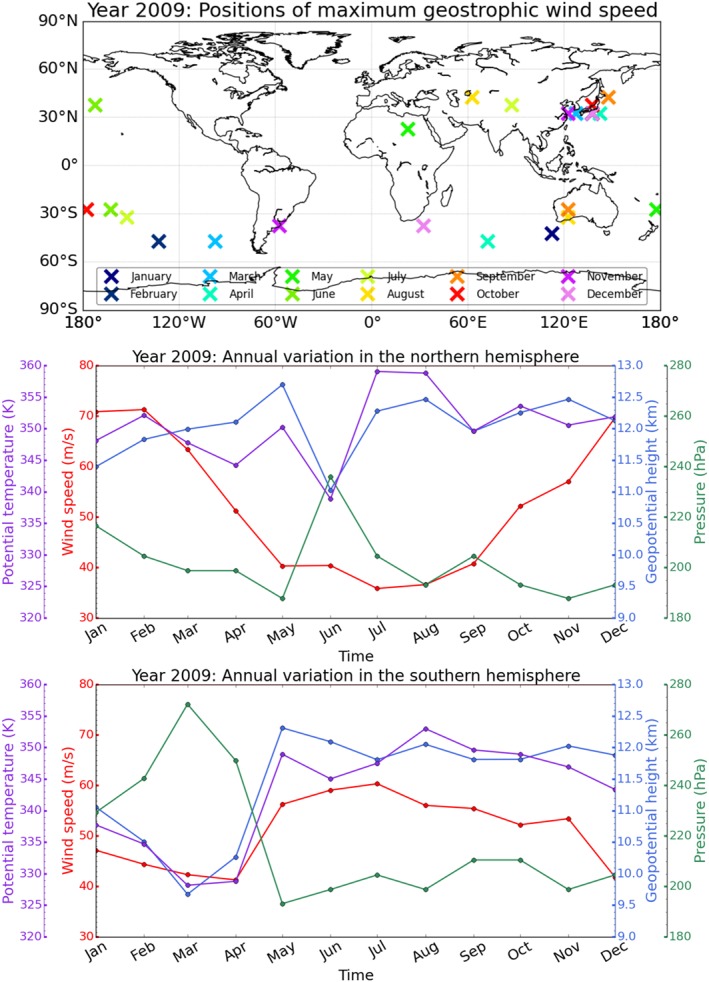
(top) Locations of Northern Hemisphere and Southern Hemisphere wind speed maxima in different months (indicated by different colors). Time series, from January 2009 to December 2009, of (middle) Northern Hemisphere and (bottom) Southern Hemisphere maximum wind speed (red), and of geopotential height (blue), pressure (green), and potential temperature (violet), at the location where the maximum wind speed has been observed.

Figure 8 reveals distinct differences of the locations of these jet stream cores between the Northern and the Southern Hemispheres. While the maximum wind speed is observed in very different longitudinal regions in the Southern Hemisphere, it is most frequently found above the west Pacific close to China in the Northern Hemisphere. Exceptions only occur during the Northern Hemisphere summer months, when wind speed is low.

The jet stream wind speed shows a distinct seasonal cycle with highest speeds in hemispheric winter. In the Northern Hemisphere hardly any seasonal cycle is noticeable for geopotential height, pressure, and potential temperature at the location of the jet stream core. Maximum wind speed is found between 11 km and 12.7 km in geopotential height, 190 hPa and 215 hPa in pressure, and 345 K and 360 K in potential temperature. An outlier in the temporal evolution of these three variables is found in June 2009, when the maximum wind speed is comparatively small (40 m s^−1^) and found in the central Pacific close to the date line.

In the Southern Hemisphere, the seasonal cycle of maximum wind speed is synchronous with geopotential height and potential temperature but asynchronous with pressure. Highest wind speeds are found between 9.7 km and 12.3 km in geopotential height, 195 hPa and 270 hPa in pressure, and 330 K and 355 K in potential temperature.

Uncertainty of these climatological wind features can be obtained from comparing them to geostrophic and actual wind features from monthly mean ECMWF analysis fields. Similar to *Scherllin‐Pirscher et al.* [2014], we find in this study that ageostrophy is the dominant error source (not separately illustrated). While maximum wind speed as well as horizontal and vertical positions of the jet stream cores are almost the same for ECMWF geostrophic winds, actual ECMWF winds in jet stream cores are slightly higher (5 m s^−1^ to 10 m s^−1^). Horizontal and vertical locations of jet stream cores are generally in good agreement, but actual wind speeds from ECMWF do not show the outlier in June 2009 in the Northern Hemisphere.

We note that the results shown are those obtained from calculating geostrophic wind on pressure levels, i.e., based on equation (9). The comparison to the results based on isentropic coordinates (not shown) reveals that they are almost identical, confirming the robust versatility of the RO data and vertical coordinate choices. A comparative disadvantage of isentropic coordinates is, however, that it is more challenging to ensure a sufficiently dense vertical spacing of the grid. In some situations, we even found the 1 K spaced tropospheric potential temperature grid to be too coarse to appropriately identify the level of maximum wind speed.

## 4 Summary and Conclusions

High‐resolution observations from radio occultation (RO) measurements provide virtually independent information on altitude and pressure. This unique property among satellite‐based observational systems is important because it ensures equivalent data quality on isohypsic (MSL altitude and geopotential height) and thermodynamic (pressure and potential temperature) vertical coordinates.

Isohypsic vertical coordinates from RO measurements are independent of atmospheric conditions. Their uncertainty is only limited by the choice of the Earth geoid model and its horizontal resolution as well as (in case of geopotential height) the accuracy of the Earth gravity model. Adequate and readily available geoid models, such as the Earth gravity models EGM96 and EGM2008, are consistent to better than 1 m accuracy. Comparison of geopotential height obtained from two independent gravity models revealed likewise very small differences in the troposphere and stratosphere (better than 2 m accuracy up to 35 km). Both models, the Joint Gravity Model (JGM‐3) as well as an approximation using the formula of Somigliana and a truncated Taylor series expansion (Appendix A), can therefore equally be used to map MSL altitude to geopotential height.

In order to provide error estimates of physical atmospheric variables from RO on isohypsic coordinates, we advanced the tropospheric part of the empirical‐analytical error model of *Scherllin‐Pirscher et al.* [2011a, 2011b] that was restricted to RO‐derived dry atmospheric variables. Due to the increasing influence of background information below 15 km in physical atmospheric variables, tropospheric observational errors of temperature and potential temperature were found to decrease about linearly toward the boundary layer, while pressure errors increase about linearly over this range.

The total climatological error, which is composed of the statistical error, (residual) sampling error, and systematic error, was found to moderately increase from the tropopause toward the boundary layer, mainly due to the increasing systematic error. The systematic error is the dominating error component at low latitudes, where atmospheric variability is, in general, small. In regions with high atmospheric variability (i.e., at high latitudes), however, the residual sampling error becomes more important than the systematic error, in line with the previous results by *Scherllin‐Pirscher et al.* [2011a, 2011b].

Using sampling error‐corrected mean climatological fields, we demonstrated the high value of vertical geolocation with RO for two exemplary applications in atmospheric dynamics. We presented the annual cycle of the tropopause location and discussed its characteristics and uncertainties related to different vertical coordinates, geopotential height, pressure, and potential temperature. We furthermore investigated climatological wind fields regarding the location and annual cycle characteristics of maximum column wind speed in jet stream cores and discussed uncertainties.

Both examples clearly reveal the power of vertical geolocation from RO due to its high vertical resolution, high accuracy and precision, global availability, and virtually independent information on altitude and thermodynamic atmospheric parameters. These are key factors to reliably determine characteristics of atmospheric levels and make RO a unique observational data set for investigating the vertical structure of the troposphere and stratosphere in any coordinate system.

## Appendix A Modeling of Geopotential Height From Geodetic Height and Gravity

### A.1

Normal gravity at the surface of the Earth's ellipsoid, *γ*(*φ*), is given by the closed formula of Somigliana [*Moritz*, 2000],

(A1) $$\gamma (\varphi )=\frac{a\gamma_{a}\overset{2}{\cos}\varphi +b\gamma_{b}\overset{2}{\sin}\varphi }{\sqrt{a^{2}\overset{2}{\cos}\varphi +b^{2}\overset{2}{\sin}\varphi }},$$

where *a* = 6378137.0 m and *b* = 6356752.3142 m are the semimajor and the semiminor axes of the Earth, respectively, and *γ*_*a*_=9.7803253359 m s^−2^ and *γ*_*b*_=9.8321849378 m s^−2^ are the equatorial and polar gravity, respectively.

In WEGC's occultation processing system (OPS) [*Schwärz et al.*, 2016], we implemented the conventional abbreviated series expansion of equation (A1) for normal gravity [*Moritz*, 2000], which reads

(A2) $$\gamma (\varphi )=\gamma_{a}\left[1+\beta \overset{2}{\sin}\varphi -f\left(\frac{5}{2}m-\frac{1}{2}f\right)\frac{1}{4}\overset{2}{\sin}2\varphi \right],$$

where *β* = (*γ*_*b*_−*γ*_*a*_)/*γ*_*a*_ is the flattening of the Earth's gravity; *f* = (*a* − *b*)/*a* is the flattening of the Earth's figure; and *m* = (Ω^2^ *a* ^2^ *b*)/(*G* *M*), where Ω = 7.2921 × 10^−5^ rad s^−1^ is the Earth's rotation rate and *G* *M* = 3986004.418 × 10^8^ m^3^/s^2^ is the Earth's gravity‐mass factor.

A frequently used Taylor series expansion for normal gravity above the ellipsoid with direction along the geodetic normal to the reference ellipsoid is [*National Imagery and Mapping Agency (NIMA)*, 2000],

(A3) $$g(\varphi ,h)\approx \gamma (\varphi )\left[1-\frac{2}{a}\left(1+f+m-2f\overset{2}{\sin}\varphi \right)h+\frac{3}{a^{2}}h^{2}\right],$$

where *h* is the ellipsoidal height.

Geopotential height *Z* above the Earth's ellipsoid is obtained from

(A4) $$Z(\varphi ,h)=\frac{1}{g_{0}}\overset{h}{\underset{0}{\int }}g(\varphi ,h^{\prime })dh^{\prime },$$

where *g*_0_=9.80665 m s^−2^ is the Earth's standard gravity. Using normal gravity above the ellipsoid, equation (A3), and applying the integration according to equation (A4), we obtain geopotential height as a function of latitude *φ* and ellipsoidal height *h*

(A5) $$\begin{array}{cc} Z(\varphi ,h) & \approx \frac{\gamma (\varphi )}{g_{0}}\overset{h}{\underset{0}{\int }}\left[1-\frac{2}{a}\left(1+f+m-2f\overset{2}{\sin}\varphi \right)h^{\prime }+\frac{3}{a^{2}}h^{\prime 2}\right]dh^{\prime }\\ =\frac{\gamma (\varphi )}{g_{0}}h\left[1-\frac{h}{a}(1+f+m-2f\overset{2}{\sin}\varphi )+\frac{h^{2}}{a^{2}}\right]. \end{array}$$

We implemented equation (A5) in WEGC's OPS. In order to map geopotential height above the ellipsoid to geopotential height above the geoid, i.e., to calculate *Z*(*λ*,*φ*,*z*) above mean sea level (MSL) as conventionally used in atmospheric sciences, we finally compute

(A6) $$Z(\lambda ,\varphi ,z)=Z(\varphi ,h)-Z(\varphi ,N_{u}(\lambda ,\varphi )),$$

where *z* is MSL altitude and *N*_u_ is the geoid undulation as introduced in section 2.2 in the main text.

Gravity information can also be obtained from Earth geopotential models such as the Joint Earth Gravity Model (JGM‐3) [*Tapley et al.*, 1996]. The use of this model in RO retrievals is described by *Leroy* [1997]. We investigate the accuracy of geopotential height from using the analytical approximation described above by comparing it to geopotential height obtained when using the JGM‐3. This comparison (see Figure A1) confirms very good agreement of the results based on the two models.

Even though the differences increase linearly with height (due to the truncation of the Taylor series when estimating gravity above the Earth's surface), differences between the results from the two models remain smaller than 1−2 m up to 35 km geopotential height. Zonal mean differences even remain well smaller than 1 m up to 40 km geopotential height.

## Appendix B RO Error Model

### B.1

The parameterized vertical model of the observational error is based on *Steiner and Kirchengast* [2005], as also followed by [*Scherllin‐Pirscher et al.*, 2011a, 2011b]. It is given by

(B1) $$s_{\mathrm{model}}(z)=\left\{\begin{array}{cc} s_{0}+q_{0}\left[\frac{1}{z^{b}}-\frac{1}{z_{\mathrm{Ttop}}^{b}}\right] & \mathrm{for}\;\;1.6\;\mathrm{km}<z\leq z_{\mathrm{Ttop}}\\ s_{0} & \mathrm{for}\;\;z_{\mathrm{Ttop}}<z<z_{\mathrm{Sbot}}\\ s_{0}\cdot \exp\left[\frac{z-z_{\mathrm{Sbot}}}{H_{S}}\right] & \mathrm{for}\;\;z_{\mathrm{Sbot}}\leq z<35\;\mathrm{km}\;. \end{array}\right.$$

where *z*_Ttop_ and *z*_Sbot_ are the top of the troposphere domain and the bottom level of the stratosphere domain, respectively, *s*_0_ is the error in the UTLS core region, *q*_0_ the best fit parameter for the tropospheric model, *b* its exponent, and *H*_S_ the stratospheric error scale height. Because of biases in RO data in the moist lower troposphere [*Sokolovskiy*, 2003], the model is only valid above the planetary boundary layer (the top of this layer is formally set to 1.6 km). The top height of the model applicability (35 km) is set due to increasing influence of background data in temperature, pressure, and potential temperature in the stratosphere.

To account for the errors' latitudinal and seasonal variations, *Scherllin‐Pirscher et al.* [2011a] and *Scherllin‐Pirscher et al.* [2011b] extended the vertical model by adding the capability to express model parameters, such as *s*_0_ and *H*_S_, in the form

(B2) $$x(\varphi ,\tau )=x_{0}+\mathrm{\Delta xf}(\varphi )\left[f_{\mathrm{\Delta x}0}+f_{\mathrm{\Delta x}s}g(\tau ,\varphi )\right]\;,$$

where *x*_0_ is the basic mean magnitude of the parameter *x* (i.e., *s*_00_ is the basic mean magnitude of the error in the UTLS core region and *H*_S0_ the basic mean magnitude of the stratospheric error scale height), Δ*x* is the maximum amplitude of latitudinal and/or seasonal variations of *x* (i.e., Δ*s*_0_ is the maximum amplitude of the variations of the error in the UTLS core region, and Δ*H*_S0_ is the maximum amplitude of the variations of the stratospheric error scale height), *f*(*φ*) accounts for latitudinal dependence, and *g*(*τ*,*φ*) for seasonal variations of *x*. *f*_Δ*x*0_ and *f*_Δ*x*s_ assign the fraction of Δ*x* that flows into latitudinal change and seasonality, respectively. Both these fraction parameters are designed to vary between zero and unity. *f*(*φ*) and *g*(*φ*) are modeled as

(B3) $$f(\varphi )=\max\left\{0,\min\left[\left(\frac{|\varphi |-\varphi_{\mathrm{\Delta x}\mathrm{lo}}}{\varphi_{\mathrm{\Delta x}\mathrm{hi}}-\varphi_{\mathrm{\Delta x}\mathrm{lo}}}\right),1\right]\right\}$$

and

(B4) g(τ,φ)=sign(φ)cos(2πτ),

where

(B5) $$\tau =\left\{\begin{array}{cc} \frac{(m-1)-m_{\mathrm{lag}}}{12} & \mathrm{for}\;\;m\in \{1,\ldots ,12\}\\ \frac{3s-m_{\mathrm{lag}}}{12} & \mathrm{for}\;\;s\in \{1,\ldots ,4\}\\ \frac{(d-15)-30.5m_{\mathrm{lag}}}{366} & \mathrm{for}\;\;d\in \{1,\ldots ,366\}\;. \end{array}\right.$$

The observational error model accounts for latitudinal and seasonal variations of the stratospheric error scale height *H*_S_ only. To model *x* = *H*_S_, we set *f*_Δ*x*0_=0 and *f*_Δ*x*s_=1 and chose *φ*_Δ*x*lo_=30° and *φ*_Δ*x*hi_=60°. Using these settings following *Scherllin‐Pirscher et al.* [2011b], the observational error strongly increases in the stratosphere at high latitudes in winter (Figure 3, bottom row). The estimated model parameter values are summarized in Table 1.

For the sampling error as well as for the systematic error, we accounted for latitudinal and seasonal variations of the error in the UTLS core region in terms of the error magnitude *s*_0_. To model the latitudinal and seasonal variations of *x* = *s*_0_ for the systematic error *s*_sysErr_, we set *f*_Δ*x*0_=1.0 and *f*_Δ*x*s_=1.0 and *φ*_Δ*x*lo_=50° and *φ*_Δ*x*hi_=60°, identical to *Scherllin‐Pirscher et al.* [2011a]. Modeling *x* = *s*_0_ for the sampling error *s*_samplErr_, we chose *f*_Δ*x*0_=0.75 and *f*_Δ*x*s_=0.25 and *φ*_Δ*x*lo_=40° and *φ*_Δ*x*hi_=90°, very similar to *Scherllin‐Pirscher et al.* [2011a]. These settings yield increased systematic and sampling errors at high latitudes poleward of *φ*_Δ*x*lo_. Furthermore, the errors are larger in hemispheric winter than in hemispheric summer.

## Appendix C Glossary of Key Terms

### C.1

We briefly define here key terms related to the vertical geolocation and error estimation discussed in the paper. The geodetic definitions given are suitable for atmospheric sciences and applications where vertical level accuracies within about 1 m can be considered highly accurate and sufficient. More detailed information is found in the geodetic specialist literature [e.g., *NIMA*, 2000; *Hofmann‐Wellenhof and Moritz*, 2006; *Torge and J. Müller*, 2012].

*Earth's ellipsoid*: Shape of the Earth's surface if it is assumed to be influenced only by the Earth's zero‐order (spherically symmetric) gravitational field and its rotation. The WGS 84 ellipsoid is defined by the Earth's major (equatorial) axis *a* = 6378137.0 m and the inverse flattening 1/*f* = 298.257223563, corresponding to a minor (polar) axis of *b* = 6356752.3 m.

*Earth's geoid*: Shape of the Earth's surface if accounting for the influence by the Earth's higher‐order gravitational field and its rotation (spherical harmonics expansion to orders beyond 300, reflecting the realistic inhomogeneous mass distribution of the Earth's crust and interior). The sum of the gravitational potential energy and centrifugal potential energy (i.e., the gravity potential energy) is the same for all points on the geoid, which represents the equipotential surface corresponding to mean sea level (MSL) of the oceans extended through the continents.

*Ellipsoidal height (or simply height)*: Height of a given point above the Earth's ellipsoid. Same as geodetic height. Usual symbol: *h*.

*Geodetic height*: Same as ellipsoidal height (see above).

*Geoid undulation*: Height difference between the Earth's geoid and ellipsoid at any given geographic location. The geoid undulation can be both positive or negative, depending on whether the geoid (MSL) surface is higher or lower than the ellipsoidal surface; globally, it deviates from the Earth's ellipsoid at most up to about 100 m. Usual symbol: *N*_u_.

*Geopotential height*: Height of a given point above the Earth's geoid (MSL) when taking account of the geographic and vertical effects of local gravity anomalies. Geopotential height can be considered as a vertical adjustment to MSL altitude (or orthometric height) such that in the adjusted height the acceleration of gravity appears globally constant (at the value of the standard acceleration of gravity *g*_0_=9.80665 m/s^2^) in the hydrostatic balance equation in the atmosphere. Usual symbol: *Z* (if emphasizing geopotential height of pressure levels, *Z*_*p*_).

*Height AGL*: Height of a given point above ground level (AGL; sometimes also abbreviated a.g.l.), i.e., height above the topographic surface of the Earth. The height AGL is equal to the orthometric height (MSL altitude) over the oceans and otherwise deviates from it by the orthometric height of the topographic surface. Usual symbol: *h*_AGL_ (if unambiguous in context also just *h*_a_).

*Isentropic coordinates*: Vertical level surfaces of constant potential temperature throughout the atmosphere. They can account for dry air only, depending only on pressure and temperature in this case, or also include humidity (virtual potential temperature). In the latter case small differences occur for significant moisture in the lower troposphere (see section 2.2). Usual symbol: *θ* (if emphasizing virtual potential temperature, *θ*_v_).

*Isobaric coordinates*: Vertical level surfaces of constant pressure (or log‐pressure or pressure altitude; see section 3.1) throughout the atmosphere. Log‐pressure and pressure altitude coordinates employ logarithmic pressure for convenience, so as to obtain a vertical scaling similar to the scaling of isohypsic coordinates. Usual symbols: *p*, log‐*p*, and *z*_*p*_.

*Isohypsic coordinates*: Vertical level surfaces of constant height or altitude (i.e., one of ellipsoidal height/geodetic height, geopotential height, and MSL altitude/orthometric height) throughout the atmosphere. Isohypsic vertical coordinates depend only on the geometric position within the Earth's coordinate frame; i.e., they do not depend on the atmospheric state. Usual symbols: *h*, *Z*, and *z*.

*Measurement height of RO observations:* Same as ellipsoidal/geodetic height; ellipsoidal (WGS 84) coordinates are commonly used as the standard coordinate frame for GNSS‐related data processing.

*MSL altitude (or altitude AMSL or simply altitude):* Height of a given point above the Earth's geoid, i.e., above mean sea level (MSL; sometimes also abbreviated AMSL, amsl, and asl). Same as orthometric height. Usual symbol: *z*.

*Orthometric height:* Same as MSL altitude (see above).

*Random statistical error:* Random error of individual measurement profiles. The random statistical error decreases when averaging over an increasing number of measurement profiles.

*Residual sampling error:* Residual error due to spatiotemporal undersampling after accounting for (i.e., subtracting) the estimated sampling error.

*Sampling error:* Error in an (RO‐derived) climatological field of an atmospheric variable (e.g., temperature) due to undersampling of the spatial and temporal variability of the field by the (sparse) geographic distribution of (RO) measurement locations. Can be reasonably estimated based on gridded atmospheric analysis fields of adequate quality (e.g., ECMWF analyses).

*Spatial resolution:* The spatial area or volume of the atmosphere, sometimes also termed resolution kernel, that contributes to a single geolocated (RO) measurement value of an individual sounding observation (RO event). For RO generally specified in terms of horizontal (along ray/across ray) and vertical resolution of RO profiles.

*Spatial sampling:* The average spatial geographic distance, usually computed for global (RO) coverage, between adjacent sounding observations (RO event locations) within a defined observing cycle (e.g., per day or month). For RO the sampling density achieved depends in particular on the number of RO receiver satellites available simultaneously in low Earth orbit and the scope of their GNSS signal tracking capabilities.

*Systematic error:* Error in the retrieved atmospheric profiles due to biases in the measurements and/or due to the retrieval processing.

*Total climatological error:* Root‐mean‐square sum of the random statistical error, the systematic error, and the residual sampling error (or the sampling error, if it is not subtracted from the climatological field).
